# Iconic Meaning in Music: An Event-Related Potential Study

**DOI:** 10.1371/journal.pone.0132169

**Published:** 2015-07-10

**Authors:** Liman Cai, Ping Huang, Qiuling Luo, Hong Huang, Lei Mo

**Affiliations:** 1 Center for Studies of Psychological Application, South China Normal University, Guangzhou 510631, China; 2 College of Education Science, South China Normal University, Guangzhou 510631, China; 3 Department of Music, Xinghai Conservatory of Music, Guangzhou, 510500, China; University Zurich, SWITZERLAND

## Abstract

Although there has been extensive research on the processing of the emotional meaning of music, little is known about other aspects of listeners’ experience of music. The present study investigated the neural correlates of the iconic meaning of music. Event-related potentials (ERP) were recorded while a group of 20 music majors and a group of 20 non-music majors performed a lexical decision task in the context of implicit musical iconic meaning priming. ERP analysis revealed a significant N400 effect of congruency in time window 260-510 ms following the onset of the target word only in the group of music majors. Time-course analysis using 50 ms windows indicated significant N400 effects both within the time window 410-460 ms and 460-510 ms for music majors, whereas only a partial N400 effect during time window 410-460 ms was observed for non-music majors. There was also a trend for the N400 effects in the music major group to be stronger than those in the non-major group in the sub-windows of 310-360ms and 410-460ms. Especially in the sub-window of 410-460 ms, the topographical map of the difference waveforms between congruent and incongruent conditions revealed different N400 distribution between groups; the effect was concentrated in bilateral frontal areas for music majors, but in central-parietal areas for non-music majors. These results imply probable neural mechanism differences underlying automatic iconic meaning priming of music. Our findings suggest that processing of the iconic meaning of music can be accomplished automatically and that musical training may facilitate the understanding of the iconic meaning of music.

## Introduction

As language, music is claimed to be able to convey meaningful information. The musical meaning mainly comprises two dimensions: emotional meaning, and iconic meaning [1–4]. Recent research has focused on exploring the mechanism by which the emotional meaning of music is understood [5–8]. The priming paradigm has been widely adopted in previous studies, with the presentation of a specific segment of music containing emotional information preceding target words of corresponding emotional meanings. Analogous to semantic priming, emotional priming effects only occur if the musical segment can convey emotional information. In a behavioral experiment, Sollberge, Rebe, and Eckstein [6] revealed a significant priming effect of musical chords on target words of similar emotional valence. Evidences from ectrophysiological studies which employed affective priming paradigms also support the idea that music can convey emotional meaning. Priming effects were found to be induced by both chord primed affective words and affective word primed chords [7]. Meanwhile, ERPs also showed comparable N400 effects: a larger N400 elicited by targets affectively unrelated to their primes, no matter in condition of chord priming word or word priming chord. Recently, Painter & Koelsch [9] demonstrated that even short musical sounds outside of a musical context are capable of conveying meaningful information, but sounds require more elaborate processing than other kinds of meaningful stimuli.

Noticed the observed N400 priming effect of music could be due to task-induced strategies (i.e. the process can be modified by the level of attention), more implicit tasks were adapted to investigate that whether the N400 effect can be automatically elicited in processing of musical meaning (i.e., attention- independent). In the electrophysiological study by Daltrozzo and Schön [10], a lexical decision task was applied. Comparing with related music-word pairs, enhanced N400 response was found for conceptual unrelated music-word pairs, suggesting the role of music in automatically conveying affective meanings. Similar results were found by Cai and colleagues [11] using Chinese words of high emotional valence. However, there have been some contradicting results. Goerlich and colleagues [12] adopted both explicit affective categorization and implicit lexical categorization tasks to study the impact of music on lexical processing. The N400 component was observed in the affective categorization but not in the lexical categorization of the same stimuli. We suppose the divergent results might derive from the difference of stimuli arousal levels or the experimental paradigms. Generally, existing results provide strong evidence of the affective priming effects of music.

Although empirical studies support the claim that music can convey its emotional meaning, few studies have focused on listeners’ experience of the iconic meaning of music. Music likely evokes certain images or situations in the listener’s mind, and if so, the iconic information of music should be significantly primed by relevant iconic words. In an ERP study conducted by Koelsch and colleagues [5], the N400 component was found in a semantic priming task, indicating a salient semantic priming effect of music. However, target words were categorized as either concrete or abstract, while the emotional valences of target words were not well counter balanced. Therefore, the significant priming effect can be alternatively explained as emotion priming. Given this, more direct evidence for iconic meaning of music is needed, which serves as the first goal our study.

The second aim of this study was to explore the role of expertise in understanding iconic meaning in music. Accumulated evidence has shown brain plasticity after long-term musical training [13–16]. A recent study examined neuroplasticity of professional musicians while performing a conceptual task (i.e., picture-word matching) and a perceptual task in an fMRI scanner [17]. They found that conceptual processing of visually presented musical instruments activates the auditory association cortex as well as adjacent areas in superior temporal sulcus and middle temporal gyrus of the right hemisphere in musicians, but not in musical laypersons. These results suggest a reorganization of conceptual representation patterns shaped by long-term musical experience. Thus, we inferred that the neuroplastic changes in musicians may facilitate the automatic processing of the iconic meaning of music. Because most of the previous studies on priming effects of musical sounds on the processing of word meaning were focused on amateur subjects, the present study examined the musical iconic meaning processing distinction between music majors and non- music majors. To avoid the explicit task-induced strategies that may impact the N400 effect, we used a lexical decision task (LDT) as described in Daltrozzo and Schön’s study [10].

We hypothesized that, first, the processing of the iconic meaning of music can be accomplished automatically. That is, N400 priming effects can be observed via implicit LDT: the iconic meaning incongruent condition would give rise to a larger N400 to targets compared to the congruent condition. Second, professional music training should facilitate the understanding of the iconic meaning of music and have an impact on the N400 effect.

## Materials and Methods

### Ethics Statement

The current study was approved by the Academic committee of the School of Psychology at South China Normal University. All participants gave written informed consent before participating in the experiments.

### Participants

Forty students from South China Normal University aged 19–24 voluntarily participated in the experiments. Twenty music majors (8 males) who had received regular professional musical training participated in Experiment 1. The other twenty students (10 males) of other majors without any professional musical training experience participated in Experiment 2. All the participants were right-handed native Chinese speakers with normal hearing and normal or corrected to normal vision. No psychiatric or neurological disorder was reported. Participants were given a small payment after the experiment.

### Materials

The experimental stimuli were created in the following steps. First, musical images were generalized into 7 categories (i.e., people, animals, plants, objects, structures, scenes, and natural phenomena) according to musicological and aesthetic qualities (Table 1 lists some examples). One hundred and eighty two-character Chinese nouns corresponding to these 7 categories were selected. All words were high or middle frequency nouns (>10 per million, according to the Modern Chinese Frequency Dictionary [18]). Second, corresponding to the these nouns, one hundred and eighty 5000 ms instrumental music excerpts without voice or lyrics were selected by three music experts. Third, 27 non-music majors used a 7-point scale to rate the extent to which the iconic meaning of the music segments and words matched. None of these participants was included in the ERP experiments and none of them had any professional musical training experience. The 120 pairs of musical segments and words that received the highest ratings (Mean = 5.28, SD = 0.54) were selected as experimental stimuli. (e.g., for the word [Swan], an excerpt of Mozart’s third violin concerto, second movement, interpreted by Anne-Sophie Mutter on violin, 1978). Fourth, 120 music-word unrelated pairs were built from these 120 matching pairs by re-matching the stimuli from every two iconic meaning irrelevant pairs. Table 2 shows some examples of related and unrelated music-noun word pairs. Fifth, in order to ask participants to complete a lexical decision task, we selected another eighty music excerpts of a similar style to the 120 excerpts mentioned above, and paired them with eighty pseudo-words as filling material. Finally, in order to avoid the repeated exposure of the musical excerpts, we created two versions of the materials. Both versions consisted of 200 music-word pairs, including 60 related pairs, 60 unrelated pairs and 80 filling pairs. We divided the 120 music-word pairs into Group A and Group B, balancing the average word frequency, the average number of character strokes and the music-word relatedness rating. One version of the materials was created by combining the music-word related pairs from group A, the unrelated pairs from group B and the filling material. The other version was created by combining the unrelated pairs from group A, the related pairs of group B and the filling material.

**Table 1:** Examples of noun words used in present study.

Categories	Examples of nouns
People	warrior lover girl clown witch king thief
animals	swan butterfly mouse bee bird wolf chicken
Plants	lily lotus cirrus forest leaf straw reef
objects	candle sponge saw glass clock cartoon crystal
structures	gate church castle tower basement maze temple
scenes	celebration dreamscape holiday wedding ruins
natural phenomena	storm moonlight brook sun winter ocean vortex

**Table 2:** Examples of the material of music excerpts and iconic meaning related or unrelated words.

Music excerpt-word			Related Excerpt References
Musical image	Word		
	matched	unmatched	
storm	storm	swan	Vivaldi: The Four Seasons, the third movement
swan	swan	storm	Mozart: The third violin concerto, the second movement
vortex	vortex	desert	Prokofiev: Romeo and Juliet—fight
desert	desert	vortex	Borodin: In the Steppes of Central Asia
church	church	brook	Respighi: Church Windows
brook	brook	church	Smetana: Vltava River
butterfly	butterfly	lover	Mozart: Concerto For Flute, Harp
lover	lover	butterfly	Bach: Air On the G String

### Experimental procedure

Experimental procedures and stimuli presentations were identical for the two experiments. Participants were asked to focus on the center of the screen throughout the trials. A music excerpt, with an average length of 5 seconds, was presented with a fixation on screen, followed by a blank inter-stimulus interval (ISI) of 400ms on average. A target word of 800ms was then presented (1° in vertical angle and 4.8° in horizontal angle), followed by a pair of question marks (duration: 1600ms), after which subjects were to decide whether the target was a word or a pseudo-word by pressing one of two keys. The association between hand side and response was counter-balanced. At the end of every trial, participants were shown XXXX for 2700ms, which directed them to relax and blink. All the stimuli were programmed and presented with E-prime 1.2 (Psychology Software Tools, Inc., Pittsburgh, PA).

### Data acquisition and analysis

EEG data were acquired with 32 bit resolution at a sampling rate of 1000 Hz by a Neuroscan, NuAmps amplifier (Compumedics Neuroscan Inc.) with 32 channel Quick Caps. The signals were on-line low-pass filtered at 100 Hz. The recorded scalp sites included FP1, FP2, F7, F8, F3, F4, FT7, FT8, T3, T4, FC3, FC4, C3, C4, CP3, CP4, TP7, TP8, T5, T6, P3, P4, O1, O2, Fz, FCz, Cz, CPz, Pz, Oz. All the electrodes were referenced online to the right mastoid, and re-referenced offline to the average of the left and right mastoid. Impedances of all electrodes were lowered to below 5kΩ before the experiment.

ERP data offline processing was performed using SCAN Edit (Neuroscan). After re-referencing and DC correction, Ocular artifacts were removed, and then a zero-phase digital filtering with 0.05–30 Hz as cut-off (slope = 24dB/octave) was applied. Thereafter, the data were divided into epochs of 1000 ms, including a 200 ms pre-stimulus baseline. A threshold of ±100μV was used for epoch rejection.

We dismissed all the ERP and behavior data obtained from music-pseudoword pairs as filters and analyzed those from music-word pairs. Based on our visual inspection and previous results [19, 20], mean amplitudes were quantified for the time window of the N400 from 260 ms to 510 ms after the target onset. To examine the distribution of N400, three different regions of interest (ROI) were defined for statistical analysis: frontal area [F3, Fz, F4, FCz], central area [C3, Cz, C4] and parietal area [CPZ, P3, Pz, P4]. Repeated measures analysis of variance (ANOVA) with the within factors being Congruency (congruent vs. incongruent) and ROI (frontal vs. central vs. parietal areas) were performed separately for the two groups, music majors and non-music majors. For further analysis of time-course changes in the N400 effect of congruency, in each experiment, the main time window was first segmented into five 50 ms sub-windows, and then, repeated measures ANOVA (Congruency×ROI) was re-conducted for each sub-window separately. To assess if there were any group differences between the musically trained students and the group of non-trained students, we performed a three factors ANOVA (Congruency×ROI×Training) within six time-windows, including the main time-window (from 260 ms to 510 ms) and five 50 ms sub-windows therein. Greenhouse-Geisser correction and Multivariate Analysis of Variance (MANOVA) were used when a statistically significant violation of the sphericity assumption was observed in Mauchly's test [21, 22]. Bonferroni correction was used for the multiple comparisons.

Response time (RT) was dismissed in behavior data analysis because of the delayed responding demand in our tests. We conducted paired-sample t-tests examining the accuracy difference between congruent and incongruent conditions, in the two groups of participants. The results of these t-tests are reported below. All statistical analyses were performed using SPSS 16.

## Results

### Behavioral data

For music majors who participated in Experiment 1, task performance (i.e., accuracy rate, or ACC) was slightly but not significantly better (*t*_19_ = 2.090, *p* = 0.050, *Cohen's d* = 0.658) in the music-word congruent condition (ACC_congruent_ = 96.99%, SD = 2.32) than in the incongruent condition (ACC_incongruent_ = 92.55%, SD = 9.25). For non-music majors who participated in Experiment 2, a similar pattern was observed, i.e., participants achieved a little higher accuracy in the music-word congruent condition (ACC_congruent_ = 95.58%, SD = 6.24) than in the music-word incongruent condition (ACC_incongruent_ = 94.42%, SD = 6.56), but the difference did not reach significance (*t*_19_ = 1.889, *p* = 0.074, *Cohen's d* = 0.182).

### ERP data

#### Experiment 1

**Fig 1:**
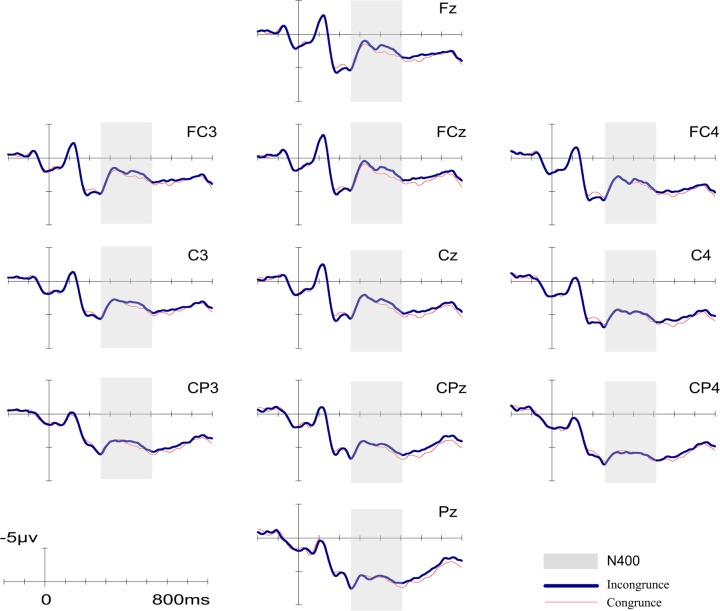
Grand-averaged ERPs in the Experiment 1 (music major group). N400 time windows are highlighted with gray markers. ERPs of related condition is shown as red thin lines and unrelated condition is shown as blue thick lines.

**Fig 2:**
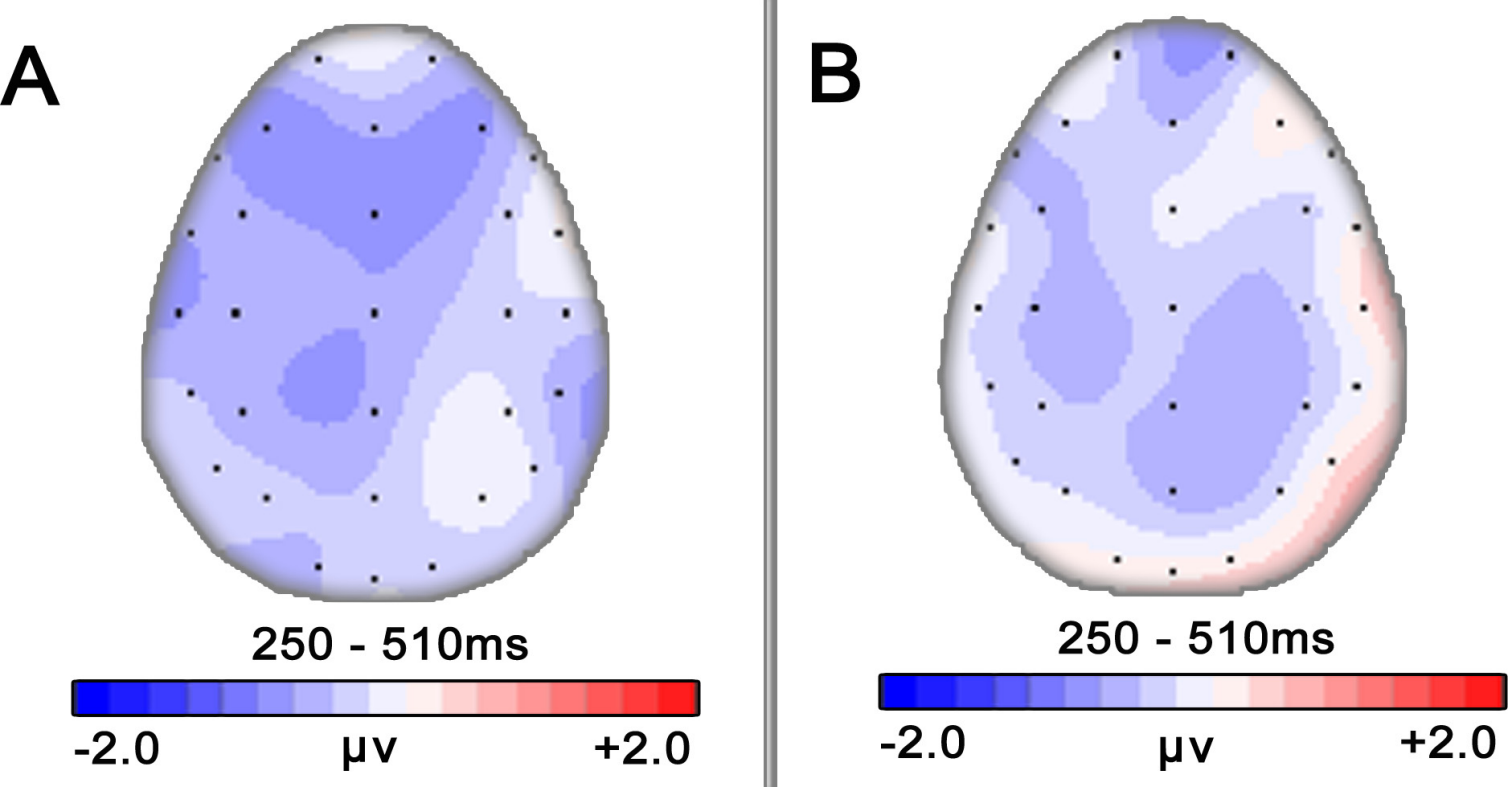
Difference-wave maps of N400(260ms~510ms) in Experiment 1 (A) and Experiment 2 (B).

**Fig 3:**
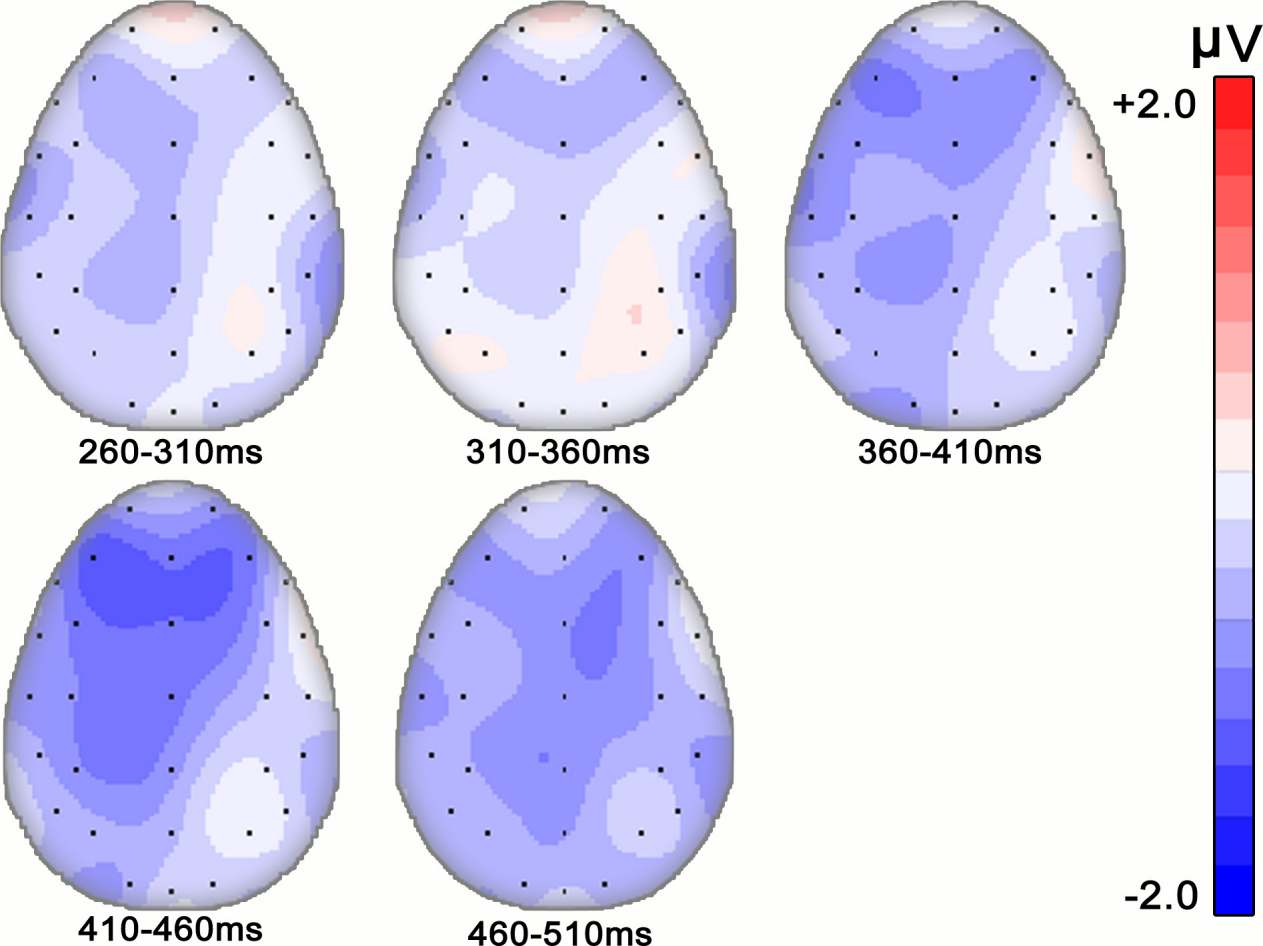
Time course of N400 effects in Experiment 1 (music major group). Difference-wave topography is shown in maps of 50ms duration from 260ms to 510ms.

Fig 1 shows the average ERPs elicited by target words when music major participants performed lexical decision tasks. In most of the electrodes, ERPs elicited from target words in both conditions began a negative-going N400 component at around 260 ms and lasted to 510 ms after target onset. In our chosen time window (260–510 ms), the results of a 2 (Congruency: congruent vs. incongruent) × 3 (ROI) repeated measures ANOVA revealed a significant main effect of congruency (*F* (1, 19) = 5.712, *p* = 0.027; η^2^ = 0.231), with larger N400 amplitude for incongruent condition than that for congruent condition (4.03±0.82 VS. 4.59±0.75), as expected. We also found a main effect of ROI (*F* (2, 38) = 8.758, *p* = 0.006; η^2^ = 0.316). Multiple comparison indicated that the mean amplitude at parietal area was significantly larger than those at anterior and central area (*ps*<0.05, both). No significant interaction was detected (*F* (4, 76) = 0.873, *p* = 0.377, η^2^ = 0.044). Fig 2a indicates the distribution of different waveforms (incongruent > congruent). To illustrate the time course changes in N400, a separate repeated measures ANOVA was performed for each 50 ms sub-window (see Fig 3). Significant N400 effects of congruency were confirmed in both sub-windows of 410–460 ms and 460–510 ms (*F* (1, 19) = 5.669, *p* = 0.028, η^2^ = 0.230 and *F* (1, 19) = 6.041, *p* = 0.024, η^2^ = 0.241, respectively), but no significant interactions were detected. Significant ROI effects were also found in every sub-windows (*ps*<0.01, both).

#### Experiment 2

**Fig 4:**
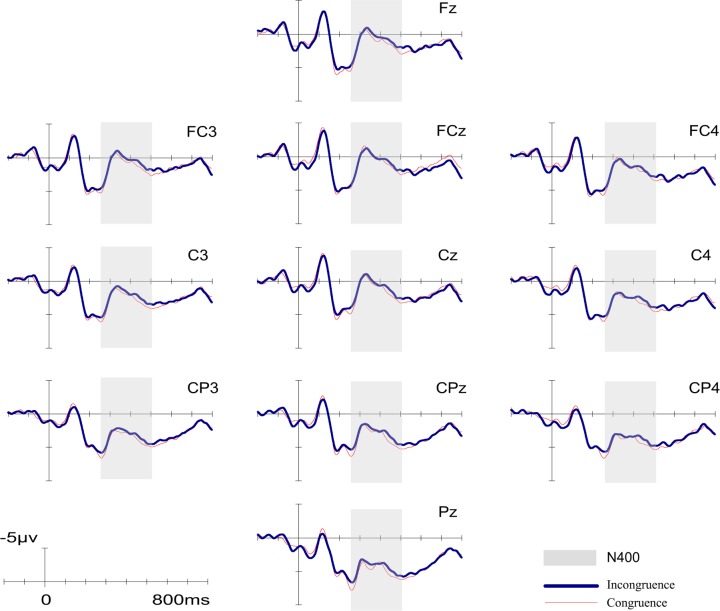
Grand-averaged ERPs in the Experiment 1 (non-music major group). N400 time windows are highlighted with gray markers. ERPs of related condition is shown as red thin lines and unrelated condition is shown as blue thick lines.

**Fig 5:**
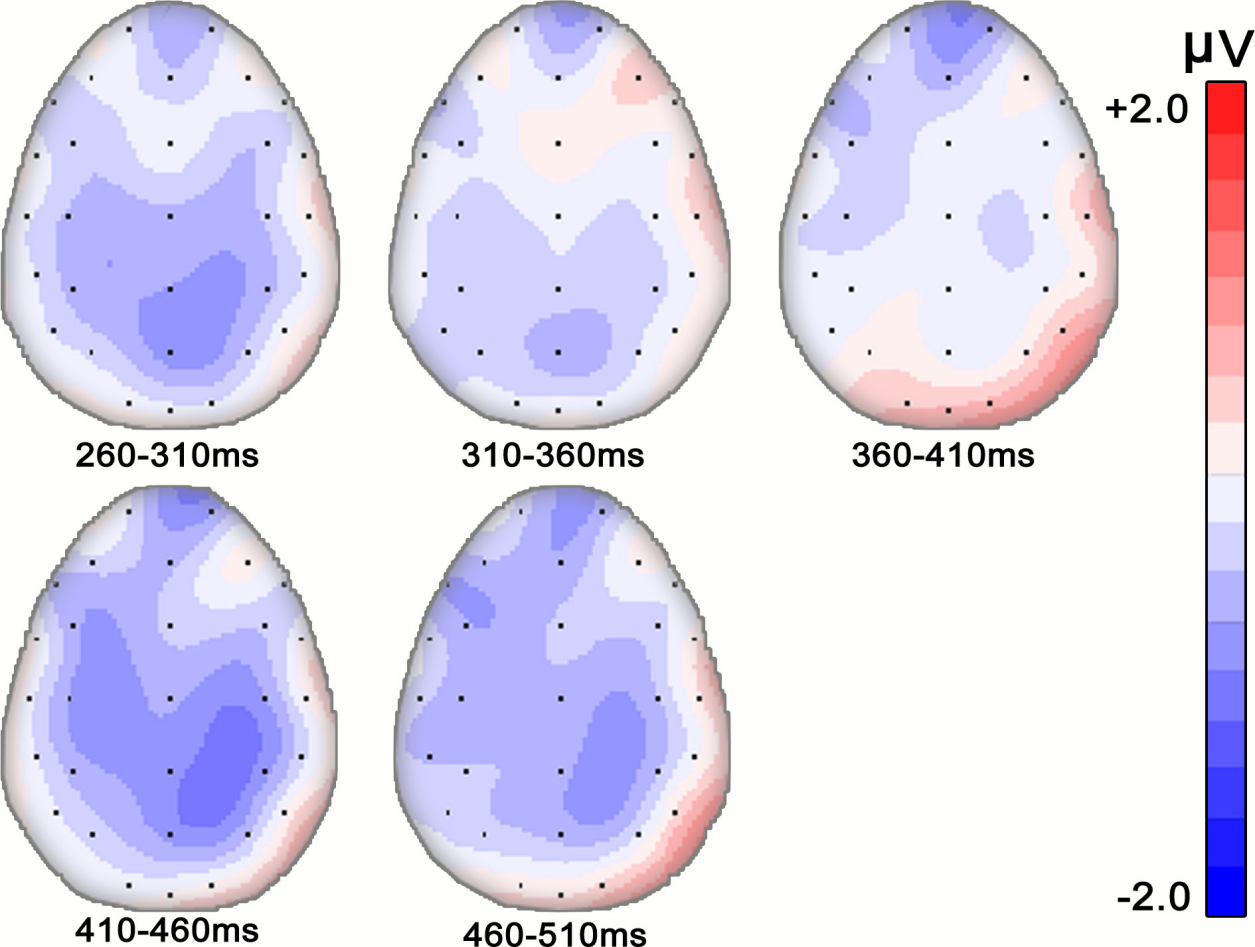
Time course of N400 effects in Experiment 2 (non-music major group). Difference-wave topography is shown in maps of 50ms duration from 260ms to 510ms.

As shown in Fig 4, for non-music majors, N400 evoked by target words started from 260 ms after the presentation onset and lasted for about 250 ms. Similar to the results of experiment 1, a significant main effect of ROI was found by repeated measures ANOVA (*F* (2, 38) = 21.172, *p* < 0.001; η^2^ = 0.527). The results of Bonferroni correction for multiple comparision analysis indicated that the mean amplitude at parietal area was significantly lager those at anterior and central area (*ps*<0.001). Moreover, the mean amplitude at central area was significantly lager than that at anterior area(*p* = 0.03). However, repeated measures ANOVA revealed neither a significant main effect of congruency (*F* (1, 19) = 2.365, *p* = 0.141, η^2^ = 0.110) nor a significant congruency by ROI interaction (*F* (2, 38) = 0.575, *p* = 0.568, η^2^ = 0.029). Fig 2b illustrates the difference waveform (incongruent > congruent) distribution. Further time course analysis was then implemented. As experiment 1, significant ROI effect was found in every sub-windows (*ps*<0.01). In the sub-window of 410–460 ms, we found a weak but not significant N400 effect of congruency (*F* (1, 19) = 3.530, *p* = 0.076, η^2^ = 0.157). Using MANOVA of Pillai's Trace, we also found a significant interaction between congruency and ROI (*F*(2, 18) = 3.893; *p* = 0.039; η^2^ = 0.302). To further examine the simple effects in this sub-window, paired-sample t-tests were conducted. We found a significant N400 effect of congruency at central area (PD_central_ = -0.70, *p* = 0.026) and a slight but not significant N400 effect at parietal area (PD_parietal_ = -0.61, *p* = 0.058). No significant effects were observed in other sub-windows. In summary, these results of non-music majors group ended up providing only partial support for our hypothesis. Time course changes of different waveforms were displayed in Fig 5.

In order to find differences in N400 effect between groups, we further conducted a set of three-way repeated measures ANOVAs (Congruency×ROI×Training). Analysis of the ERP data in the time window of 260–510 ms for both groups revealed significant main effects of congruency (*F* (1, 38) = 7.971, *p* = 0.008; η^2^ = 0.170) and ROI(*F* (2, 76) = 24.160, *p* < 0.001; η^2^ = 0.389). Multiple comparison analysis revealed that the mean amplitude for incongruent condition was smaller than that for congruent condition(3.22±0.55 VS. 3.67±0.50). The multiple comparisons also indicated that the mean amplitude of pictorial area was significantly larger than that of central and anterior area (*ps*<0.001, both), and the mean amplitude of central area was significantly larger than that of anterior area (*p* = 0.004). Contrary to our expectations, we did not find either a significant three-way interaction between congruency, ROI, and training, or any significant two-way interactions (for all tests *p*>0.1). The time course analysis showed significant main effects of congruency in the sub-windows of 410–460 ms and 460–510 ms (*F* (1, 38) = 9.194, *p* = 0.004, η^2^ = 0.195 and *F* (1, 38) = 6.363, *p* = 0.016, η^2^ = 0.143, respectively). In every sub-windows, significant ROI effects were found(*ps*<0.001, both), which was consistent with our previous results. We also found slight but not significant three-way interactions between congruency, ROI, and training in sub-windows of 310–360 ms and 410–460 ms (*F* (2, 76) = 2.726, *p* = 0.100, η^2^ = 0.067 and *F* (1, 38) = 2.450, *p* = 0.120, η^2^ = 0.061, respectively).

## Discussion

Using different techniques of composition, composers write music describing not only inner feeling, but also objects in the outside world. Because music is a form of art to describe the world, it is reasonable that when people appreciate music, corresponding situations or images will be evoked, inducing the specific iconic meaning of the music [23]. Despite the fact that the iconic meaning of music is as important as the emotional meaning of music, surprisingly very little research has been carried out to address the question of whether music can activate meaningful iconic representations. In the present study, we adopted an implicit paradigm similar to Daltrozzo and Schön's [10] and also controlled the iconic meaning of the priming music, to inspect whether the processing of iconic meaning can be automatically achieved.

We obtained different results from our behavior data and our ERP data. Our behavioral data showed that there was slight but not significant difference in the accuracy rate between word-music congruent and incongruent conditions, irrespective of the professional level of the participants. An implicit task may have an influence on the efficiency of the priming effects. For example, using several hundred microseconds of music excerpts, Goerlich [12] found a significant N400 priming effect on emotional words only when the task was related to emotion. Another study [10] with an implicit task and longer duration of excerpt found significant N400 effects but negative priming effects in behavior results. In view of this, we recently repeated Daltrozzo and Schön’s study [10] using a set of music excerpts of longer duration of about 5 seconds [11]. Both the behavior and ERP results in that study showed the significant emotional priming effects on adjective word decision. In the present study, we also used about 5 seconds of music excerpts for priming. However, we did not find significant priming effects in the behavior results, which indicated that it might be harder to automatically convey the iconic meaning of music than the emotional meaning, and that longer excerpt duration might be necessary to promote the iconic meaning priming effects of music.

Although the priming effect did not reach full statistical significance in the behavior results, the ERP data did give some evidence of the iconic meaning of music. In Experiment 1, our ERP results revealed a significant N400 main effect of congruency in the time window of 260–510 ms. In Experiment 2, no significant N400 main effect of congruency was observed in the same time window. However, we did find a significant interaction of congruency and ROI in 410–460 ms, suggesting a partial distribution of the N400 component in this sub-window. In summary, our results support our first hypothesis that the processing of the iconic meaning of music can be accomplished subconsciously.

Our previous study [11] on the role of music in automatically conveying affective meanings showed an earlier and longer N400 effect (from 250 ms to 550 ms) compared with the results of the present work. The earlier N400 effect of the affective priming of music partly agrees with the view that emotion is a primary pathway to establishing meaning in music [5, 7, 24]. However, there is evidence supporting the idea that conceptual and emotional processes of environmental sounds are functionally independent [25]. More studies are necessary to confirm whether emotional processing is a first step in musical iconic meaning processing.

Recently, Steinbeis and Koelsch investigated whether there is a training factor that affects musical affective priming effects on the processing of word meaning [7, 8]. They found no significant difference between a trained musician group and the untrained group either in behavior results or N400 effects, including latency and distribution. In the present study, we performed a three factors ANOVA (Congruency×ROI×Training) to investigate whether there were different N400 effects between the music major group and non-major group. Similar to the results of Steinbeis and Koelsch’s work, there was no significant difference between these two groups. However, it is worth noting that in the present work, significant N400 effect of congruency was found in the time windows of 260–510 ms for music majors, whereas for non-music majors, the main effect was not found significant, implying that music majors may have an advantage in musical iconic meaning processing; time course analysis also revealed a trend for a group difference in the topographic distribution of N400 effect in sub-window of 310–360 ms and of 410–460 ms. In particular, in the sub-window of 410–460 ms, the peak amplitude of different waveforms was concentrated in bilateral frontal areas for music majors (Fig 3) whereas for non-music majors, the peak amplitude of different waveforms was concentrated in central-parietal areas (Fig 5).

The tendency for the groups to differ in N400 effects might imply a neural mechanism difference in musical iconic meaning processing between the musically trained group and the untrained group. Earlier research proposed that rule violation in both language and music would lead to bilateral or lateralized frontal negative components [26–32]. Recently, research on rules and memory representation [33] in music revealed that rule violations elicit an early right-lateralized anterior-central negativity, while memory violations elicit a posterior negativity considered as an N400. To summarize, we propose that music majors construct the iconic meaning of music on the basis of musical structural organization, whereas non-music majors represent the iconic meaning of music based on associations with some specific musical experience.

There are two limitations in the present study. One limitation of our results is that, although we found a tendency toward a group difference in N400 effect between the musically trained and untrained group, the difference did not reach a significant level. The lexical decision task may be responsible for the floor effect. A semantic decision task, a biological properties judgment task for example, is a candidate to improve the effect size in further investigation. The second limitation of this work is that we used 5 sec duration of music excerpts as priming material, which excludes the possibility of using ERP data to study the iconic meaning priming effects of words on music. Further studies are needed to employ a word-music priming paradigm and directly investigate the information processing of musical iconic meaning with fMRI or fNIR techniques.

In conclusion, the present study confirms our first hypothesis that processing of the iconic meaning of music can be accomplished automatically. Our data, to some extent, also support our second hypothesis that the professional musical training increases the sensitivity of iconic meaning in music, implying possible differences of underlying mechanisms of musical processing between music majors and non-majors, except for the absence of significantly different N400 effect between groups. Hence, we propose the need for further research to test whether, for professional musicians, semantic processing is accomplished more automatically and is more dependent on the structural organization of music than non-professionals.
